# Physical Exercise Affects Adipose Tissue Profile and Prevents Arterial Thrombosis in *BDNF* Val66Met Mice

**DOI:** 10.3390/cells8080875

**Published:** 2019-08-11

**Authors:** Leonardo Sandrini, Alessandro Ieraci, Patrizia Amadio, Marta Zarà, Nico Mitro, Francis S. Lee, Elena Tremoli, Silvia Stella Barbieri

**Affiliations:** 1 Centro Cardiologico Monzino IRCCS, 20138 Milano, Italy; 2 Dipartimento di Scienze Farmacologiche e Biomolecolari, Università degli Studi di Milano, 20133 Milano, Italy; 3 Department of Psychiatry, Weill Cornell Medical College of Cornell University, New York, NY 10065, USA

**Keywords:** BDNF, Val66Met polymorphism, adipose tissue, adipogenesis, arterial thrombosis, physical exercise

## Abstract

Adipose tissue accumulation is an independent and modifiable risk factor for cardiovascular disease (CVD). The recent CVD European Guidelines strongly recommend regular physical exercise (PE) as a management strategy for prevention and treatment of CVD associated with metabolic disorders and obesity. Although mutations as well as common genetic variants, including the *brain-derived neurotrophic factor (BDNF)* Val66Met polymorphism, are associated with increased body weight, eating and neuropsychiatric disorders, and myocardial infarction, the effect of this polymorphism on adipose tissue accumulation and regulation as well as its relation to obesity/thrombosis remains to be elucidated. Here, we showed that white adipose tissue (WAT) of humanized knock-in BDNFVal66Met (BDNF^Met/Met^) mice is characterized by an altered morphology and an enhanced inflammatory profile compared to wild-type BDNF^Val/Val^. Four weeks of voluntary PE restored the adipocyte size distribution, counteracted the inflammatory profile of adipose tissue, and prevented the prothrombotic phenotype displayed, per se, by BDNF^Met/Met^ mice. C3H10T1/2 cells treated with the Pro-BDNFMet peptide well recapitulated the gene alterations observed in BDNF^Met/Met^ WAT mice. In conclusion, these data indicate the strong impact of lifestyle, in particular of the beneficial effect of PE, on the management of arterial thrombosis and inflammation associated with obesity in relation to the specific BDNF Val66Met mutation.

## 1. Introduction

Despite the huge growth in knowledge and advances in the prevention and treatment of cardiovascular disease (CVD), this pathology is still the leading cause of morbidity and mortality in the world and is predicted to reach 23.3 million by 2030 [1]. It is well known that an important modifiable risk factor for CVD mortality and morbidity is represented by excessive weight [2], and several follow-up studies demonstrated that a body mass index (BMI) >25 (>75th percentile based on percentile curves of BMI in the US reference population) is associated with a higher mortality rate [3,4]. Excessive body weight may influence CVD through its effect on risk factors such as hypertension, glucose intolerance, and dyslipidemia and may contribute through not already identified mechanisms [5]. In overweight and obese patients, adipose tissue accumulation is associated with a low-grade inflammatory profile and a higher secretion of cytokines and chemokines in the circulation compared to normal weight people [6]. The resulting subclinical inflammation is associated, among others, with hypercoagulability and increased thrombotic risk due to the enhanced platelet and leukocyte numbers and reactivities [7,8,9].

International guidelines, including 2016 European Guidelines on CVD prevention in clinical practice [10], strongly recommended regular physical exercise (PE) as management for the prevention and treatment of CVD, in particular when related to obesity and metabolic disorders. Regular PE reduces adipose-derived systemic inflammation, improves endothelial function, decreases platelet and leukocyte activation, and halts the progression of coronary stenosis in both obese and normal-weight individuals [8,11,12,13,14].

Starting from the discovery that several rare forms of obesity, called monogenic obesity, result from a mutation in single genes primarily located in the leptin–melanocortin pathway [15,16], recent evidence has identified additional selected genes associated with obesity, providing that the genetic background can play a pivotal role in causing or triggering susceptibility to the pathology when associated with environmental factors such as overeating and PE reduction [17,18]. Of note, *brain-derived neurotrophic factor* (*BDNF*) is included among these genes. Genome-wide association studies (GWAS) have shown a strong association between the *BDNF* locus and anorexia nervosa, bulimia nervosa [19], or obesity [20,21]. Indeed, it is well known that *BDNF* plays an important role in energy metabolism food intake and weight control [22,23].

In this context, the common human *BDNF* Val66Met variant through reduction of the activity-dependent secretion and signaling of mature BDNF, is associated not only to neuro-psychiatric disorders [24] and CVD [25] but also to eating disorders and obesity in humans [26,27,28,29,30]. Interestingly, a knock-in mouse carrying the human *BDNF* Val66Met polymorphism has a significantly higher body weight than wild-type littermates [31], associated with a proinflammatory and prothrombotic phenotype [25]. The frequency of the Met allele has a wide range of values: in Asians, Met allele frequency is nearly 50% heterozygous, while is about 20%–30% homozygous [32,33]. In the Caucasian population the Met allele is less frequent, with a frequency of 20%–30% heterozygous and only about 4% homozygous [33,34].

The aim of the present study was to investigate the relationship between the *BDNF* Val66Met polymorphism, obesity, and thrombosis, by analyzing the adipose tissue profile in BDNF^Met/Met^ mice, and to evaluate the ability of PE to affect adipose tissue and reduce the prothrombotic phenotype in *BDNF* Val66Met knock-in mice. Finally, in vitro studies were performed to investigate the functional relevance of *BDNF* Val66Met polymorphism on adipogenesis.

## 2. Materials and Methods

### 2.1. Mice

All experiments were performed in adult (3–4 months old) wild-type BDNF^Val/Val^ and BDNF^Met/Met^ littermate mice, generated by Chen Z-Y et al. [31]. Only male mice were used to avoid the potential impact of hormones involved in the ovarian cycle in adipose tissue present in female mice. All experiments were approved by the National Ministry of Health-University of Milan Committee and of DGSA (12/2015 and 349/2015). Surgical procedures were performed in mice anesthetized with ketamine chlorhydrate (75 mg/kg; Intervet, Segrate, Milan, Italy) and medetomidine (1 mg/kg; Virbac, Milan, Italy). Mice were housed under standard conditions (20–22 °C, 12 h light/dark cycle, light on at 7 a.m.) with water and food ad libitum. All efforts were made to minimize animal distress and to reduce the numbers of animals used in this study.

### 2.2. Voluntary Physical Exercise (PE) Protocol

Mice underwent voluntary PE protocol as previously described [35,36]. Briefly, BDNF^Val/Val^ and BDNF^Met/Met^ mice were weighed and allocated randomly into running (RUN) and control (sedentary, SED) groups in cages equipped with or without running wheels, respectively, for 4 weeks with free access to food and water. Four sedentary control mice were housed in a standard polypropylene mice cage. Four runner mice were housed in standard polypropylene rat cages, with free access to two running wheels (12 cm diameter and 5.5 cm width). The greater dimensions of cages for runner mice were necessary for an adequate setup of running wheels. Running wheels were connected to an electronic counter, and the total distance ran was recorded daily. The average distance ran by a single mouse was calculated by dividing by 2 the total distance recorded per wheel (two running wheels × cage × four mice). The average distance ran by a single mouse, in our model, was comparable with the average distance reported by others [35,36,37,38].

### 2.3. Arterial Thrombosis Model

Experimental arterial thrombosis was induced as previously described [39]. Briefly, the left carotid artery of anesthetized mice was freely dissected, and a flow probe (model 0.7 VB, Transonic System, Ithaca, NY, USA) connected to a transonic flowmeter (TransonicT106) was used to measure blood flow. When blood flow was constant for 7 min (at least 0.8 mL/s), a strip of filter paper (Whatman N°1) soaked with FeCl_3_ (7% solution; Sigma-Aldrich, Saint Louis, MO, USA) was applied for 3 min, and the flow was recorded for 30 min. An occlusion was considered to be total and stable when the flow was reduced by >90% from baseline until the 30 min observation time.

### 2.4. Whole Blood Counts

Blood was collected by cardiac venipuncture into 3.8% sodium citrate (1:10 vol:vol) from anesthetized mice, and white blood cells and platelets were counted optically.

### 2.5. Platelet–Leukocyte Aggregate Analysis

Platelet/leukocyte aggregates were analyzed as previously described [40]. Briefly, citrated blood was stimulated with 5 µM ADP (Sigma-Aldrich, Saint Louis, MO, USA) for 5 min, red blood cells were lysed by FACS Lysing solution, and samples were stained with anti-CD45 and anti-CD41 and analyzed by flow FACS “Novocyte 3000”. A minimum of 5000 events were collected in the anti-CD45^+^ gate.

### 2.6. Cell Culture, Treatment, and Differentiation

The C3H10T1/2 cell line has been used to evaluate the effect of different compounds on adipogenesis processes, as previously shown [41,42,43]. C3H10T1/2 cells (ThermoFisher Scientific, Paislay, Scotland, UK) were cultured in DMEM medium supplemented with 100 U/mL penicillin (Gibco, Rodano, Milan, Italy), 100 µg/mL streptomycin (Gibco, Rodano, Milan, Italy) and 10% FBS at 37 °C in 5% CO_2_/95% air atmosphere. Cells were plated in 6-well plates at a concentration of 3.5 × 10^4^ cells/mL, and when they reached 80% confluence (day –2), they were treated with 10 ng/mL of ProBDNFVal or ProBDNFMet synthetic peptide (Alomone Labs, Jerusalem, Israel) [44,45,46] to simulate the kinetics of *BDNF* expression occurring in physiological conditions during adipogenesis [47]. Forty-eight hours later (day 0), cells were treated with adipogenic commitment mix (5 µg/mL insulin, 2 µg/mL dexamethasone, 0.5 mM IBMX, and 5 µM rosiglitazone; all from Cayman Chemical, Arcore, Italy). Insulin (5 µg/mL) was added at days 3, 5, and 7, and complete differentiation of the cells was reached at day 9.

### 2.7. Adipogenesis Evaluation by Flow Cytometry and Oil-Red-O

After ProBDNFVal or ProBDNFMet treatment, C3H10T1/2 cells were analyzed during adipogenesis by flow cytometry, as previously described [48]. Briefly, at days 3, 5, and 9, cells were harvested in ice-cold PBS, analyzed by flow cytometry, and, according to granularity (SSC-H), were divided into four categories that correlated with the increased level of cell lipid accumulation after adipogenic commitment. In particular, noninduced cells were detected in the R1 gate, while cells with increasing granularity were identified in the regions from R2 to R4.

Oil-Red-O staining was performed as already described [49] on day 9. Lipid content was quantified as absorbance at a wavelength of 518 nm using a Tecan Infinite M1000 plate reader spectrophotometer (TECAN, Männedorf, Switzerland).

### 2.8. Quantitative Real-Time Polymerase Chain Reaction (RT-qPCR)

Total RNA was isolated from mouse adipose tissue or C3H10T1/2 cells with TRIzol Reagent (Sigma-Aldrich, Saint Louis, MO, USA) and a Direct-zol RNA extraction kit (Zymo Research, Irvine, CA, USA) according to the manufacturer’s instructions. One microgram of RNA was reverse-transcribed using an iScript Advanced cDNA Synthesis Kit (Bio-Rad Laboratories, Segrate, Milan, Italy).

Samples of cDNA were incubated in 15 µL Luna^®^ Universal qPCR mix containing the specific primers and fluorescent dye SYBR Green (New England Biolabs, Pero, Milan, Italy). RT-qPCR was carried out in triplicate for each sample on the CFX Connect real-time System (Bio-Rad Laboratories, Segrate, Milan, Italy) as previously described [39]. Gene expression was analyzed using parameters available in CFX Manager Software 3.1 (Bio-Rad Laboratories, Segrate, Milan, Italy). qPCR was then carried out using the primer sequences shown in Table S1. In particular, the expression of genes relevant in adipogenesis, inflammation, and the *BDNF* pathway were assessed (*Pparγ, C/ebp-α and C/ebp-β, Adipoq, Fabp4, Adra2a, Il-6, Mcp-1, Tnf-α, Tgf-β, Pai-1, Tf, CD163, CD80, Sorl1, Sirt1, Bdnf, TrkB* full and *TrkB-T1*).

### 2.9. Adipose Tissue Histology and Quantification of Adipocyte Size and Number

Immunocytochemistry and the analysis of adipocytes were performed in inguinal (ingWAT) and epididymal (epiWAT) white adipose tissue. Tissues were fixed overnight in 4% formalin, embedded in paraffin, cut at 5 μm, and mounted on polarized slides [50]. Five sections at three different levels along the whole length of epiWAT for each animal were analyzed. In particular, the mean values for each group were obtained from a total of 90 sections (5 sections × 3 points × 6 animals/group). The tissue contiguous to the epididymis were excluded from the analyses since its structure is different from that of general adipose tissue [51].

The number and size of adipocytes were evaluated in hematoxylin and eosin stained sections by counting five 5× microscopic fields for each tissue sample using the ImageJ-Macro Adipocytes Tool. Images were taken with a Zeiss Axioskop (Carl Zeiss, Milan, Italy) equipped with an intensified charge-coupled device (CCD) camera system (Photometrics, Tucson, AZ, USA).

### 2.10. Statistical Analysis

Statistical analyses were performed with GraphPad Prism 7.0 and SAS versus 9.4 software (SASA Institute, Cary, NC, USA). Data were analyzed by Student’s t-test, two-way or three-way ANOVA with or without repeated measures for main effects of genotype and treatment or time and stimuli, as reported in every graph, followed by a Bonferroni post hoc analysis as appropriate. When data distribution was not normal, the variables were included in the analyses after logarithmic transformation. Values of *p* < 0.05 were considered statistically significant. Data are expressed as mean ± SEM.

## 3. Results

### 3.1. Characterization of the White Adipose Tissue Depots in BDNF^Met/Met^ Mice

**Figure 1:**
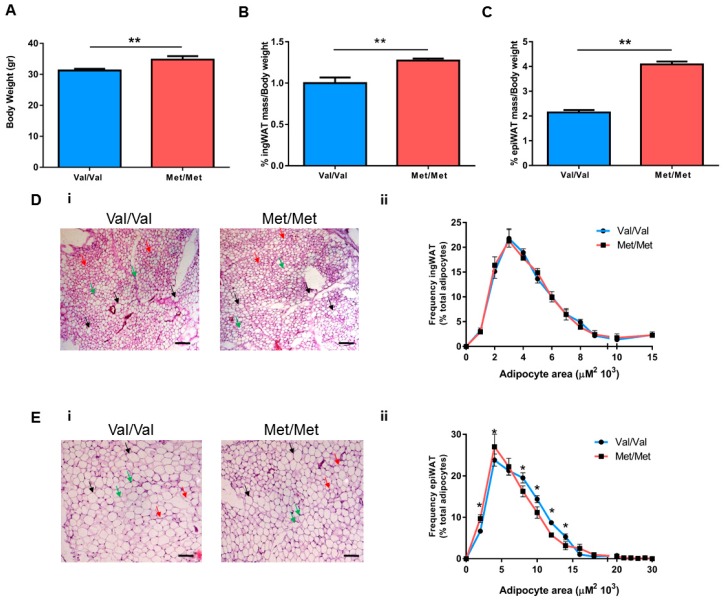
Characterization of white adipose tissue depots in BDNF^Val/Val^ and BDNF^Met/Met^ mice. (**A**) Body weight, percentage of (**B**) inguinal (ingWAT) and (**C**) epidydimal (epiWAT) white adipose tissue on total mouse body weight. (i) Representative hematoxylin and eosin (H&E) staining images and (ii) analysis of the frequency distribution of adipocyte sizes in (**D**) ingWAT and (**E**) epiWAT. Size bar: 100 µm. Black arrow: large adipocytes, green arrow: medium adipocytes, and red arrow: small adipocytes. Data are expressed as mean ± SEM. *n* = 6 mice/group. (**A**–**C**) Student’s t-test and (**D**,**E**) two-way ANOVA followed by Bonferroni post hoc analysis. * *p* < 0.05, ** *p* < 0.01.

As previously shown, BDNF^Met/Met^ mice have a significantly greater body weight compared to wild-type BDNF^Val/Val^ littermates (Figure 1A). In addition, we observed that the percentage of both inguinal white adipose tissue (ingWAT) and epididymal white adipose tissue (epiWAT) on total body weight were significantly enhanced in BDNF^Met/Met^ mice compared to BDNF^Val/Val^ (Figure 1B,C).

The histological examination of adipose depots revealed no difference in the frequency distribution of adipocyte sizes in ingWAT, while the BDNF^Met/Met^ mice showed enrichment in small-size and a reduction in middle-size adipocytes in the epiWAT when compared to BDNF^Val/Val^ (Figure 1D,E).

**Figure 2:**
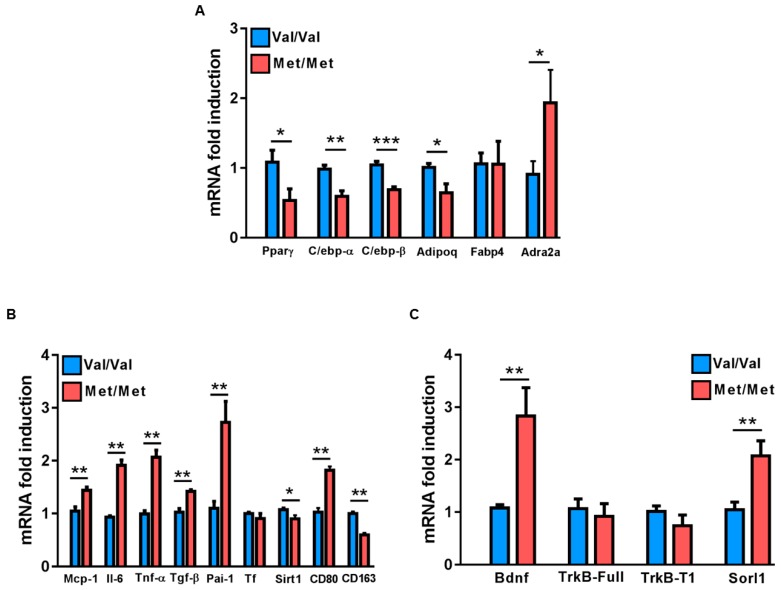
Gene expression profile of epidydimal white adipose tissue (epiWAT) in BDNF^Val/Val^ and BDNF^Met/Met^ mice. mRNA levels of genes related to (**A**) adipogenesis, (**B**) inflammation, and (**C**) BDNF/TrkB pathway in epidydimal white adipose tissue (epiWAT) of BDNF^Val/Val^ and BDNF^Met/Met^ mice. Data are expressed as mean ± SEM. *n* = 6 mice/group. Student’s t-test. * *p* < 0.05, ** *p* < 0.01, and *** *p* < 0.005.

Then, the molecular signatures underlying the distinct morphological features of the epiWAT were investigated. Mutant mice had significantly lower levels of *Pparγ, C/ebp-α* and *C/ebp-β* genes involved in the adipogenic program, as well as *Adipoq*, but a similar expression of *Fabp4* compared to BDNF^Val/Val^ mice (Figure 2A). Interestingly, the *BDNF* Val66Met polymorphism affected also the expression of *Adra2a*, *Sirt1*, and *Sorl1*, genes involved in both adipose tissue energy balance and adipocyte morphology (Figure 2A–C).

In addition, a significant increase in the expression of Il-6, Tnf-α, Tgf-β, Mcp-1, and Pai-1 in BDNF^Met/Met^ mice compared to BDNF^Val/Val^ was found, whereas similar levels of TF between the two groups were found (Figure 2B). The enhanced inflammatory profile of BDNF^Met/Met^ epiWAT was associated with a greater expression of CD80, an M1 inflammatory macrophage marker, and with a reduction of CD163, an alternatively activated M2 macrophage marker (Figure 2B).

Finally, BDNF^Met/Met^ mice had a higher *BDNF* mRNA level in epiWAT, whereas no differences in the expression of both *TrkB*-full length and the truncated isoform *TrkB-T1* were found (Figure 2C).

### 3.2. Evaluation of the Role of Mutant BDNF Val66Met Protein on Adipogenesis

Next, in vitro studies were performed to investigate the role of the BDNF Val66Met protein on adipogenesis. Pre-confluent C3H10Ts1/2 murine mesenchymal stem cells were exposed to ProBDNFVal or to ProBDNFMet synthetic peptides before inducing the adipocyte differentiation program. Synthetic peptide treatment did not affect cell number and morphology (Figure S1).

**Figure 3:**
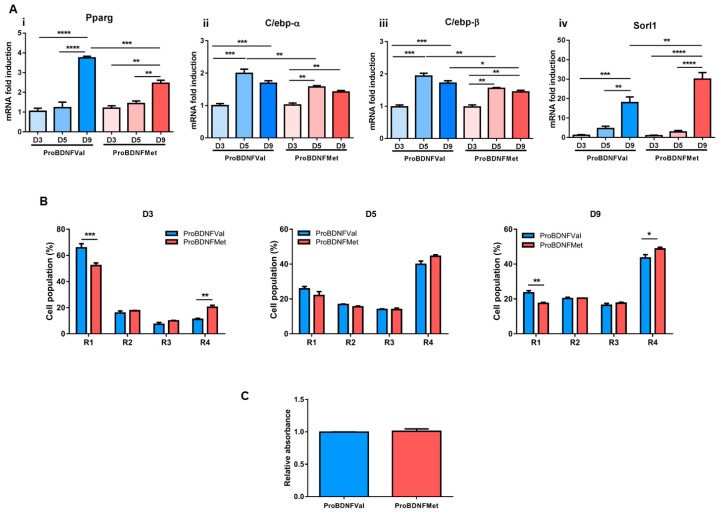
Effect of proBDNFMet on adipogenic differentiation of C3H10T1/2 cells. (**A**) mRNA levels of (i) *Pparγ*, (ii) *C/ebp-α*, (iii) *C/ebp-β,* and (iv) *Sorl1*. (**B**) Percentage of different cell populations based on their granularity profile analyzed by flow cytometry (R1: noninduced, R2-R3: growing granularity, and R4: high granularity) at day 3 (D3), day 5 (D5), and day 9 (D9) of differentiation, and (**C**) Oil-Red-O staining absorbance measurement in C3H10T1/2 cells. Data are expressed as mean ± SEM. *n* = 5 independent experiments/group. (**A**) Two-way ANOVA followed by Bonferroni post hoc analysis. (**B**,**C**) Student’s t-test. * *p* < 0.05, ** *p* < 0.01, *** *p* < 0.005, and **** *p* < 0.001.

Notably, gene expression analysis at late (day 9) stages of differentiation showed that pretreatment with the peptide carrying the Met mutation determined a significant down-regulation of adipogenic genes, including *Pparγ*, *C/ebpα* and *C/ebpβ* mRNA levels (Figure 3A). In addition, ProBDNFMet treatment decreased the percentage of cells with low granularity (noninduced; R1) and increased those with high granularity (R4) both at 3 and 9 d post-induction (Figure 3B and Figure S2). However, at day 9, as provided by the oil-red-O staining, a similar accumulation of lipid droplets was detected in both samples (Figure 3C).

In this experimental condition, among the genes that were previously modulated in epiWAT of BDNF^Met/Met^ mice, only *Sorl1* was enhanced by ProBDNFMet treatment at late stages of differentiation (day 9) (Figure 3A and Figure S3).

### 3.3. Effect of Physical Exercise (PE) on Adipose Tissue Phenotype of BDNF Val66Met Mice

According to international cardiovascular guidelines [10] that recommend regular PE as management for the prevention and treatment of CVD, we evaluated the potential beneficial effect of PE on adipose tissue and on prothrombotic phenotypes in *BDNF* Val66Met knock-in mice.

**Figure 4:**
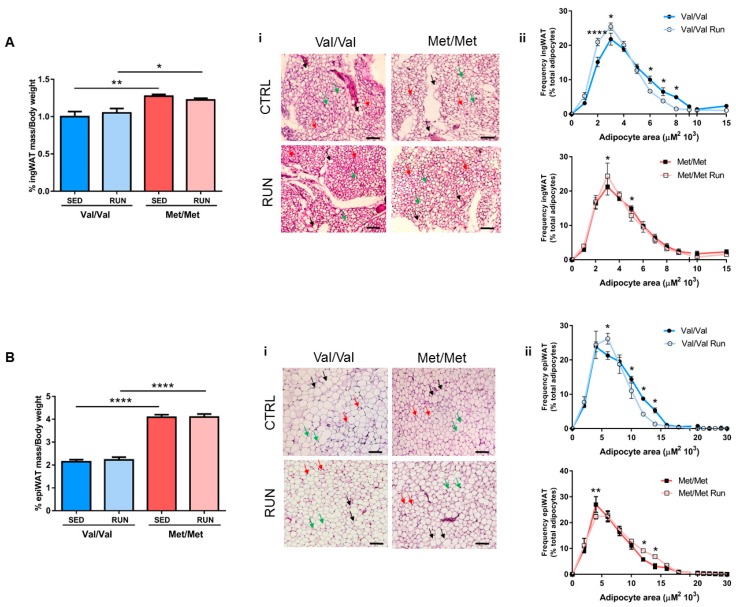
Impact of voluntary physical exercise (PE) on epiWAT morphology. (**A**) Inguinal (ingWAT) and (**B**) epidydimal (epiWAT) white adipose tissue on total mouse body weight. (i) Representative hematoxylin and eosin (H&E) staining images and (ii) analysis of the frequency distribution of adipocyte sizes in (**A**) ingWAT and (**B**) epiWAT. Size bar: 100 µm. Black arrow: large adipocytes, green arrow: medium adipocytes, and red arrow: small adipocytes. Data are expressed as mean ± SEM. *n* = 6 mice/group. Two-way ANOVA followed by Bonferroni post hoc analysis. * *p* < 0.05, ** *p* < 0.01, and **** *p* < 0.001.

BDNF^Val/Val^ and BDNF^Met/Met^ mice underwent 4 weeks of voluntary PE in cages equipped with a running wheel. As previously reported [35], no difference in the daily running distance was found between BDNF^Val/Val^ and BDNF^Met/Met^ mice (BDNF^Val/Val^: 6.676 ± 0.720 Km/d and BDNF^Met/Met^ 6.657 ± 0.602 Km/d; *p* = 0.9837) in our experimental setting. In addition, we showed that PE did not affect the percentage of ingWAT and epiWAT on the total body weight in both BDNF^Val/Val^ and BDNF^Met/Met^ mice, compared to sedentary mice, whereas the morphology of adipose depots was modified as provided by histological analyses (Figure 4).

PE induced a change in the profile of the frequency distribution of adipocyte sizes in the ingWAT of both genotypes; however, this effect was more evident in BDNF^Val/Val^ than in BDNF^Met/Met^ mice (Figure 4A).

Interestingly, in the epiWAT, BDNF^Val/Val^ running mice displayed a significant enrichment in small-size adipocytes and a reduction in medium-size ones compared to sedentary mice, whereas BDNF^Met/Met^ mice showed an opposite trend, even if less marked (Figure 4B).

**Figure 5:**
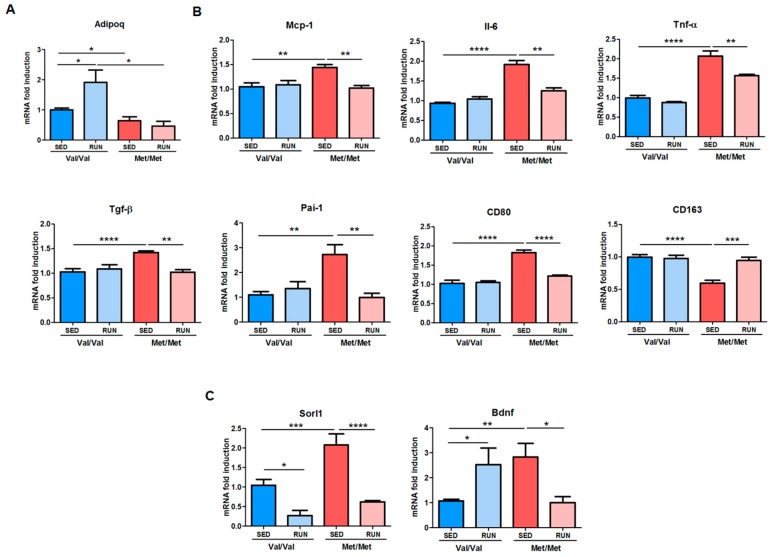
Impact of voluntary physical exercise (PE) on the gene expression profile of adipose tissue isolated from BDNF^Val/Val^ and BDNF^Met/Met^ mice. (**A**) Adipogenesis, (**B**) inflammation, and (**C**) BDNF/TrkB pathway related to mRNA levels in epiWAT of sedentary and running BDNF^Val/Val^ and BDNF^Met/Met^ mice. Data are expressed as mean ± SEM. *n* = 6 mice/group. Two-way ANOVA followed by Bonferroni post hoc analysis. * *p* < 0.05, ** *p* < 0.01, *** *p* < 0.005, and **** *p* < 0.001.

Notably, PE strongly influenced the gene expression profile of epiWAT. In particular, in BDNF^Val/Val^, 4 weeks of PE enhanced mRNA levels of *Adipoq*, whereas it did not modify the expression of genes involved in the adipogenic program (Figure 5A and Figure S4) and in inflammation compared to the sedentary mice. In BDNF^Met/Met^ mice, PE was not sufficient to affect the expression of adipogenic genes, but it was sufficient to improve the inflammatory profile, decreasing the expression of Il-6, Tnf-α, Tgf-β, Mcp-1, and Pai-1, and to switch M1/M2 macrophage polarization, reducing the expression of CD80 and increasing the expression of CD163, (Figure 5B).

In addition, the expression of *Sorl1* was markedly reduced by PE in both BDNF^Val/Val^ and BDNF^Met/Met^ mice, whereas *Adra2a* and *Sirt1* were only slightly, but not significantly, decreased in BDNF^Met/Met^ running mice (Figure 5C and Figure S4).

Conversely, PE modulated the *BDNF* expression in the two groups of mice. In particular, *BDNF* mRNA levels increased in BDNF^Val/Val^ running mice and reduced in BDNF^Met/Met^ running mice when compared to their respective sedentary controls (Figure 5C). Of note, the expression of both *TrkB* full length and the *TrkB-T1* isoform were slightly, but not significantly, increased in both groups of mice after PE (Figure S4).

### 3.4. Effect of Physical Exercise (PE) on the Pro-Thrombotic Phenotype in BDNF^Met/Met^ Mice

Finally, we investigated the ability of 4 weeks of PE to improve the prothrombotic phenotype already observed in BDNF^Met/Met^ [25], in terms of platelet and leukocyte aggregates and FeCl_3_-induced arterial thrombosis.

**Figure 6:**
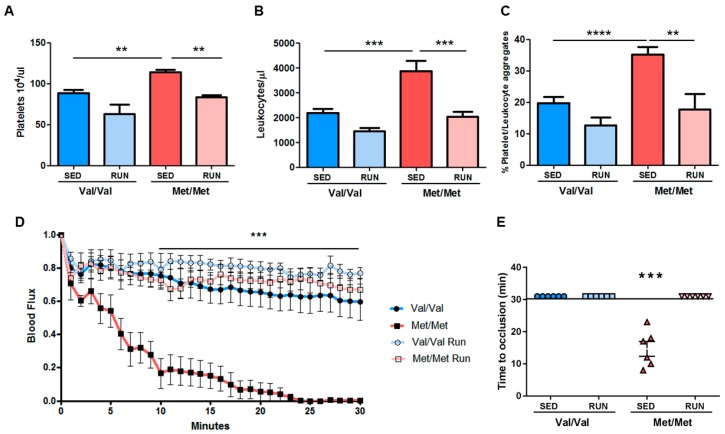
Effect of voluntary physical exercise (PE) on the prothrombotic phenotype of BDNF^Val/Val^ and BDNF^Met/Met^ mice. Numbers of circulating (**A**) platelets and (**B**) leukocytes. (**C**) Percentage of platelet/leukocytes in whole blood analyzed by flow cytometry. Arterial thrombosis induced by topical application of FeCl_3_ to the carotid artery: (**D**) blood flow and (**E**) time to occlusion measured in sedentary and running BDNF^Val/Val^ and BDNF^Met/Met^ mice. *n* = 6 mice/group. (**A**–**C** and **E**) Two-way ANOVA followed by Bonferroni post hoc analysis. (**D**) Three-way ANOVA with repeated measures followed by Bonferroni post hoc analysis. ** *p* < 0.01, *** *p* < 0.005.

As previously shown, in the BDNF^Met/Met^ mice there was a higher number of circulating blood cells, a higher platelet activation state, and enhanced arterial thrombosis [25]. PE restored the physiological number of platelets and leukocytes, and the natural percentage of platelet/leukocyte aggregates in response to ADP in BDNF^Met/Met^ mice, without affecting significantly these parameters in BDNF^Val/Val^ mice (Figure 6A–C).

Application of FeCl_3_ to the carotid artery reduced the blood flow in all BDNF^Met/Met^ sedentary mice, leading to a stable occlusion in 100% of mice, whereas only a slight reduction was observed in BNDF^Val/Val^ mice. Of note, PE ameliorated arterial thrombosis, preventing completely the occlusion of the carotid artery in BDNF^Met/Met^ mouse group (Figure 6D). In addition, no statistical differences were observed among sedentary BNDF^Val/Val^ mice and running BNDF^Val/Val^ and/or running BDNF^Met/Met^ mice in terms of carotid artery occlusion (Figure 6D). In line with these data, total occlusion (flow reduction >90%) was reached only in sedentary BDNF^Met/Met^ mice after an average time of 15 min (Figure 6E).

Overall, these data show that a paradigm of 4 weeks of voluntary PE is able to prevent the prothrombotic phenotype of BDNF^Met/Met^ mice.

## 4. Discussion

Although mutations, as well as genetic variants, including *BDNF* Val66Met polymorphism, have been associated with increased body weight and eating disorders in both human and animal models [19,20,21,22,23,31,35,52,53,54,55], the factors and mechanisms involved in the development of obesity in presence of the *BDNF* Met homozygosity remain to be elucidated. It is only known that *BDNF*-to-*TrkB* signaling is an important downstream target of MC4R-mediated signaling involved in the regulation of energy balance and food intake [55,56,57].

Using a knock-in *BDNF* Val66Met mouse model, here we confirmed that BDNF^Met/Met^ mice had a higher body weight when compared to BDNF^Val/Val^ [31], and we showed that this increase was related to the enhanced percentage of epiWAT and ingWAT. In particular, adipocytes from epiWAT of mutant mice had a different size distribution, with an enrichment in the percentage of small-sized adipocytes. The presence of small adipocytes in epiWAT of BDNF^Met/Met^ might trace back to hyperplasia or expansion of the small cell population, which are mechanisms of defense that the adipose tissue can undergo in obesity after a threshold of hypertrophy is reached [58,59,60]. This hypothesis is also supported by the higher expression of *Adra2a* and *Sorl1* found in epiWAT of BDNF^Met/Met^. Indeed, overexpression of *Adra2a* in animal models has been associated with adipose tissue hyperplasia [61]. In addition, it is well known that the activation of *Adra2a* has an antilipolytic effect, and the increased alpha/beta adrenoreceptor ratio as well as the gain of function mutations of *Adra2* have been associated with obesity in humans [62,63,64]. Similarly, upregulation of the expression of *Sorl1*, which encodes for the protein Sorla, has been related to reduced lipolytic activity in adipocytes [65], and GWAS analyses have associated *Sorl1* with obesity in humans and in mouse models [21,66], suggesting its key role in metabolic diseases.

The adipose tissue accumulation found in BDNF^Met/Met^ mice was accompanied by a higher expression of the M1 proinflammatory marker CD80, of the monocyte chemoattractant protein-1 (Mcp-1) and of the mediators of inflammation such as Pai-1, Tnf-alpha, and Il-6, which is in line with the well-established paradigm that overweight and obesity are related to adipose tissue inflammation [67]. In addition, the higher levels of these inflammatory transcripts, concomitant with the lower expression of *Adipoq* measured in the epiWAT of BDNF^Met/Met^ mice and the higher number of circulating leukocytes and platelets as well as their activation state, might well summarize the relationship between adipose tissue inflammation and thrombosis. Indeed, the inflammatory profile of adipose tissue in obese subjects as well as the increased presence of these proteins in the circulation have a direct role in the onset and progression of the pathology [68,69,70], enhancing platelet activation and ability of leukocytes to produce, in turn, inflammatory factors such as Il-6, Tnf, and Cox-2 [9,68,71,72,73,74]. All these findings thoroughly summarize data obtained in human adipose tissue samples. Indeed, a positive correlation between proinflammatory cytokines, including IL-6, TNF-α and MCP-1, and adipocyte size was found. Interestingly, the small adipocytes expressed anti-inflammatory factors such as IL-10 and IL-8 [75].

Of note, the reduced levels of *Pparγ* along with those of adiponectin found in *BDNF* mutant mice might also contribute to the observed adipose tissue inflammation. It is well known that *PPARγ*, alongside the role of master regulator of adipogenesis, is also involved in the regulation of adipose tissue inflammation. In particular, it was demonstrated that *PPARγ* downregulates inflammatory adipokines in WAT. Specifically, *PPARγ* activation downregulates the expression of inflammatory markers such as MCP-1 and TNFα and, thus, reduces inflammation in activated macrophages [56,76,77,78]. Moreover, *PPARγ* activation induces adiponectin expression, thus further contributing to the reduction of chronic inflammation [79].

Remarkably, *BDNF* expression was markedly greater in epiWAT of mutant mice, supporting our hypothesis that the *BDNF* Val66Met polymorphism contributes to adipose tissue pathophysiology.

Indeed, studies performed using *BDNF*-(si)RNA-mediated knockdown in the 3T3 cell line showed a reduced adipogenic differentiation ability, supporting the hypothesis that *BDNF* expression is of functional relevance for adipogenesis. In addition, it was reported that *BDNF* expression is dramatically downregulated during adipocyte differentiation, and mature adipocytes only marginally contribute to the production of BDNF in the adipose tissue [80].

Interestingly, we showed that the treatment of C3H10T1/2 cells with Pro-BDNFMet before cell commitment well recapitulated the expression profile of genes that were found altered in the epiWAT of mutant mice. Pro-BDNFMet reduced *Pparγ* and upregulated *Sorl1* expression, and it increased the percentage of mature adipocytes evaluated in the flow cytometry analysis, suggesting a direct role of the *BDNF* Val66Met polymorphism in the regulation of adipogenesis. However, Pro-BDNFMet was not able to affect *Adipoq* and *Adra2a* as well as *Pai-1* expression, leading us to hypothesize a more complex process that may involve the fraction stromal vascular cells. Indeed, it is suggested that mesenchymal progenitor/stem cells, preadipocytes, endothelial cells, pericytes, T cells, and macrophages, and not mature adipocytes, are the main source of adipokines and PAI-1 in adipose tissue. Of note, the stromal vascular fraction in adipose tissue increases with an increasing degree of obesity [81].

Adipose tissue accumulation represents an independent and modifiable risk factor for CVD [5], and regular PE was recently recognized and strongly recommended as a valuable management strategy for the prevention and treatment of CVD and metabolic disorders from the European Guidelines of cardiology [10,82].

In the present study, we provide evidence that, in mutant BDNF^Met/Met^ mice, four weeks of PE was sufficient to change epiWAT morphology and the inflammatory profile with a concomitant reversion of the prothrombotic phenotype. In particular, the change in adipose tissue morphology observed in BDNF^Met/Met^ running mice was accompanied with a reduction in *Sorl1* and *Adra2a* expression, thus suggesting that PE might improve the metabolic profile of mutant mice, ultimately affecting lipolysis [65,83,84].

The beneficial effect of PE has been provided in animal studies and human trials, showing an impact on both systemic [14,85] and specific reduction of visceral fat mass [86,87], protecting against chronic inflammation-associated disease [88]. Several mechanisms have been proposed to explain the beneficial anti-inflammatory effect of PE. By affecting AMPK and PGC-1α pathways, PE decreases mitochondrial dysfunction and reduces oxidative stress [89,90], with the consequent reduction of proinflammatory adipokines released from the visceral fat mass. Moreover, PE increased production of anti-inflammatory molecules from skeletal muscle and leukocytes [91]. PE decreases Toll-like receptors on monocytes and macrophages, thus preventing their infiltration into adipose tissue and inducing the M1 to M2 macrophage switching to limit macrophage M1 polarization [88].

In line with this evidence, we showed that PE in BDNF^Met/Met^ mice reduced the levels of inflammation mediators, induced a switch in macrophage polarization, and decreased the number of circulating leukocytes and platelets, modifications that, in turn, occur to improve the prothrombotic phenotype observed in mutant mice. Interestingly, for the first time, we provide evidence that PE influenced differently the expression of *BDNF* in the two genotypes, increasing and decreasing its levels in BDNF^Val/Val^ and BDNF^Met/Met^, respectively. These results might be related to the intrinsic adipose tissue morphology of BDNF^Val/Val^ and BDNF^Met/Met^ mice, suggesting a strong relationship between adipocyte dimension and *BDNF* levels. In fact, the great number of small adipocytes was associated with high levels of *BDNF* (e.g., sedentary BDNF^Met/Met^ and running BDNF^Val/Val^), and conversely, low levels of transcript were measured in epiWAT when the mean adipocyte dimension was higher (e.g., sedentary BDNF^Val/Val^ and running BDNF^Met/Met^). The different involvement of the stromal vascular cell fraction in sustaining the adipocyte turnover, as well as the potential contribution of the peripheral nervous system, might explain the different mRNA levels of *BDNF* detected in our experimental setting [92,93,94]. In this regard, the inability of PE to enhance *BDNF* transcripts in the central nervous system of mutant mice [35] might have important consequences on the levels of BDNF in the peripheral nervous system, thus affecting their levels in epiWAT. Interestingly, it is worth mentioning that, contrary to data presented here related to CVD, the *BDNF* Val66Met polymorphism impairs the beneficial neurobiological changes induced by physical exercise in mice [35].

## 5. Conclusions

Cardiovascular disease still represents the first cause of mortality worldwide, and obesity is a well-known modifiable risk factor for this pathology. Of note, PE is highly recommended to manage the prevention and treatment of CVD and obesity, showing beneficial cardiometabolic effects.

In human subjects, the *BDNF* Val66Met polymorphism is known to be related to adipose tissue accumulation and cardiovascular risk.

Interestingly, our in vitro data well support the role of Pro-BDNFMet in adipogenesis, in line with data obtained in the BDNF^Met/Met^ WAT mice.

Taking advantage of a mouse model carrying the human *BDNF* Val66Met polymorphism, we showed that 4 weeks of voluntary physical exercise was sufficient to induce positive morphological changes and reduce the inflammatory profile of the adipose tissue.

These beneficial effects might be the bases of the observed reduction in the prothrombotic phenotype detected in this animal model. Future studies are required to assess this relationship.

These data indicate the strong impact of lifestyle, in particular the beneficial effect of PE, on the management of arterial thrombosis and obesity-associated inflammation in relation to genetic mutations that predisposes one, per se, to these pathologies. Nevertheless, human studies need to support these results.

## Supplementary Materials

The following are available online at https://www.mdpi.com/2073-4409/8/8/875/s1, Figure S1: Cell number and morphology are not altered by ProBDNFVal and ProBDNFMet treatment. Figure S2: Representative flow cytometry graphs showing gate selected for cell granularity analyses at day 3, 5 and 9. Figure S3: Evaluation of the functional relevance of BDNF Val66Met protein on C3H10T1/2 cells adipogenic differentiation. Figure S4: Impact of voluntary physical exercise on gene expression profile of adipose tissue. Table S1: Primers sequences of the analyzed genes. Table S2: F and P values referred to each graph analyzed by Two-way ANOVA or Three-way ANOVA.
