# Supplementary material for: Physical Exercise Affects Adipose Tissue Profile and Prevents Arterial Thrombosis in BDNF Val66Met Mice
Source: Cells. 2019 Aug 11;8(8):875. doi: 10.3390/cells8080875 (PMC6721716; doi:10.3390/cells8080875)
Supplement: Supplementary file 1 [file cells-08-00875-s001.zip › Supplementary/Figure S1.pdf]

A

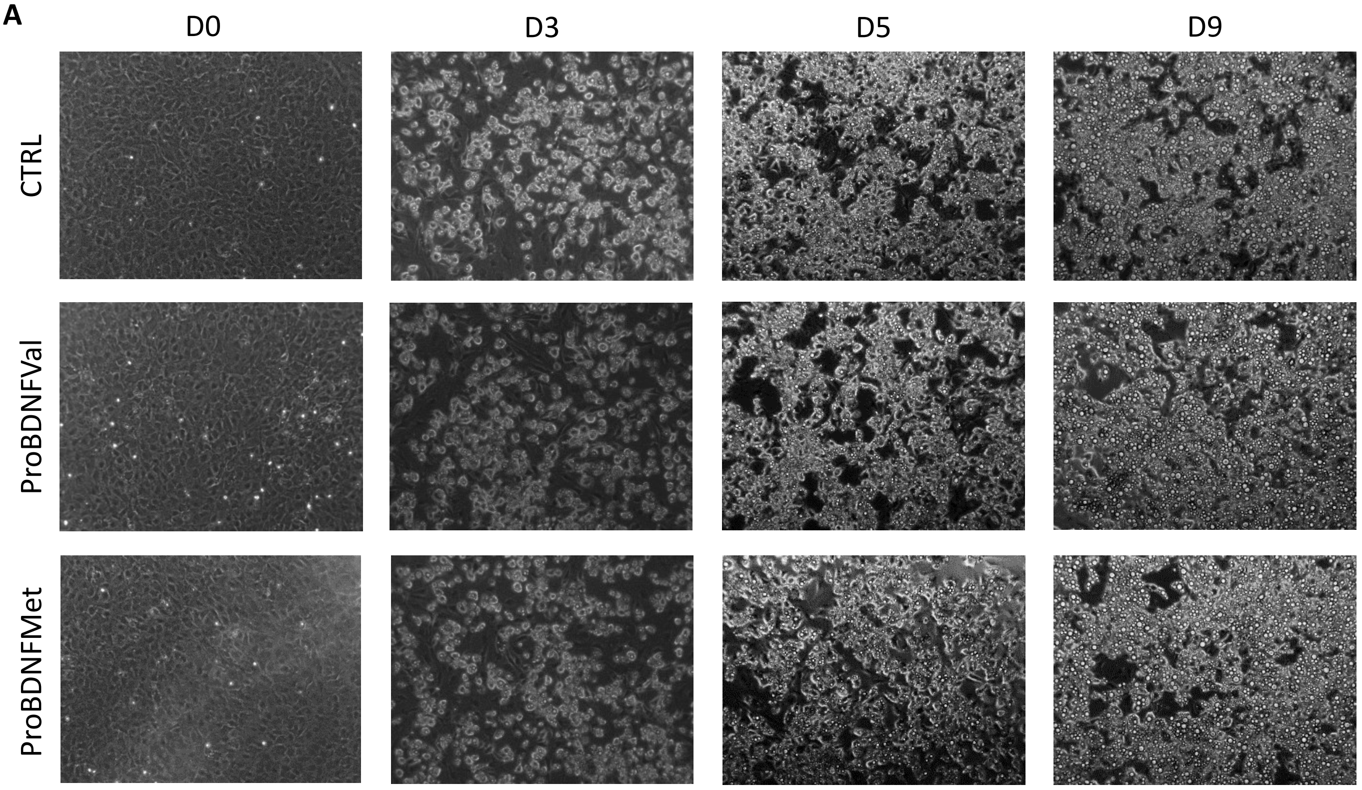

B

	**CTRL**	**ProBDNFVal**	**ProBDNFMet**	**P value**
**D0**	298337±14913	300617±15581	290793±14213	0.7140
**D3**	293687±15349	291947±17485	293270±17323	0.9912
**D5**	291943±15906	288717±13068	292050±16976	0.9560
**D9**	291157±19035	293520±15363	292357±19944	0,9875

Figure S1. Cell number and morphology are not altered by ProBDNFVal and ProBDNFMet treatment.
(A) Contrast-phase microscope images non-treated (CTRL) and treated with ProBDNFVal or ProBDNFMet peptides C3H10T1/2 cells at different time points during adipogenesis. (B) Table reporting total number of cells/well retrieved from flow-cytometry analyses at different time points during C3H10T1/2 cell differentiation. n = 5 independent experiments/group.One-way ANOVA.
