# Supplementary material for: Physical Exercise Affects Adipose Tissue Profile and Prevents Arterial Thrombosis in BDNF Val66Met Mice
Source: Cells. 2019 Aug 11;8(8):875. doi: 10.3390/cells8080875 (PMC6721716; doi:10.3390/cells8080875)
Supplement: Supplementary file 1 [file cells-08-00875-s001.zip › Supplementary/Figure S2.pdf]

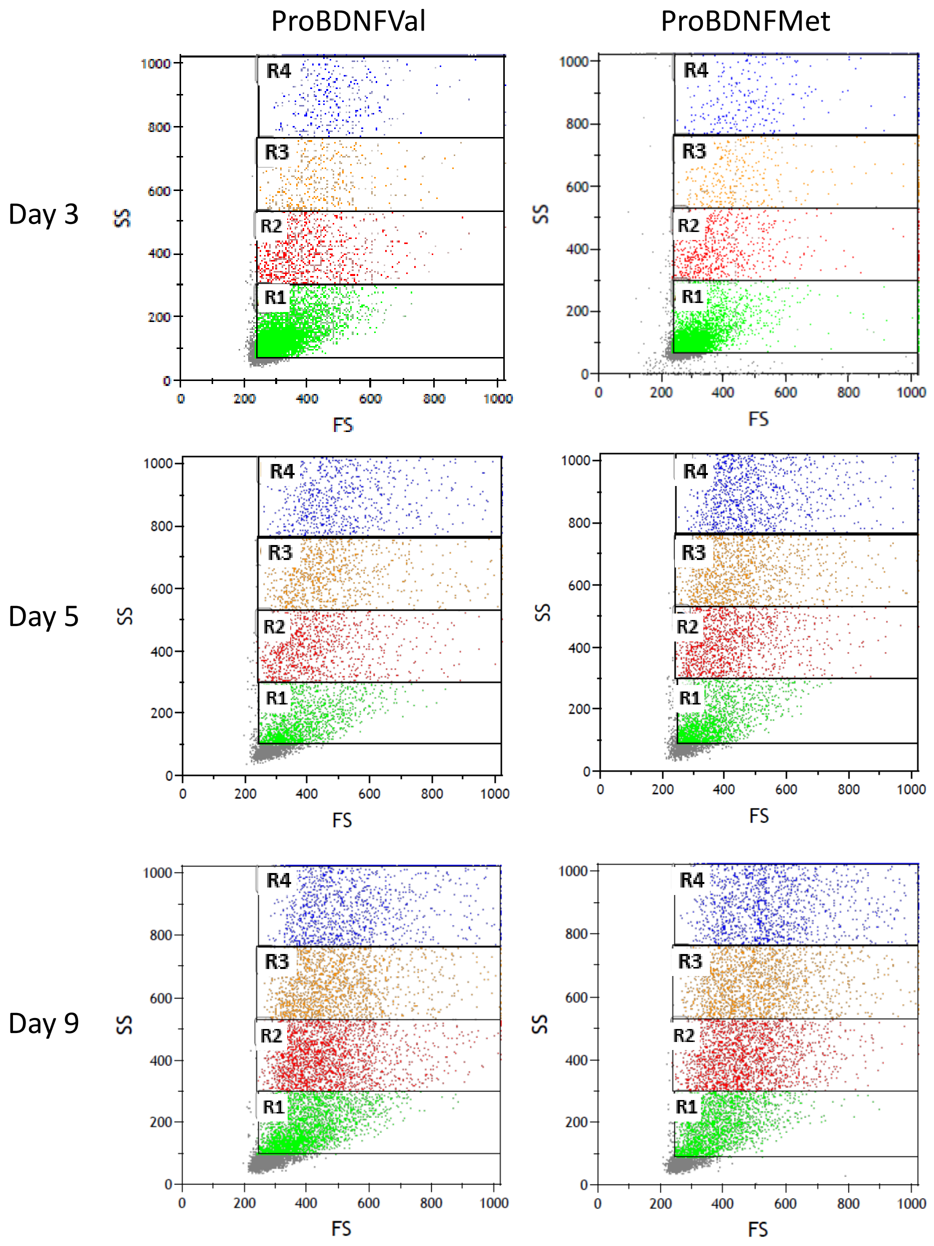

Figure S2. Representative flow cytometry graphs showing gate selected for cell granulatiry analyses at day 3, 5 and 9. Non-induced cells were detected into the R1 gate, while cells with increasing granularity were identified in the regions from R2 to R4. n = 5 independent experiments/group.
