# Supplementary material for: Physical Exercise Affects Adipose Tissue Profile and Prevents Arterial Thrombosis in BDNF Val66Met Mice
Source: Cells. 2019 Aug 11;8(8):875. doi: 10.3390/cells8080875 (PMC6721716; doi:10.3390/cells8080875)
Supplement: Supplementary file 1 [file cells-08-00875-s001.zip › Supplementary/Figure S3.pdf]

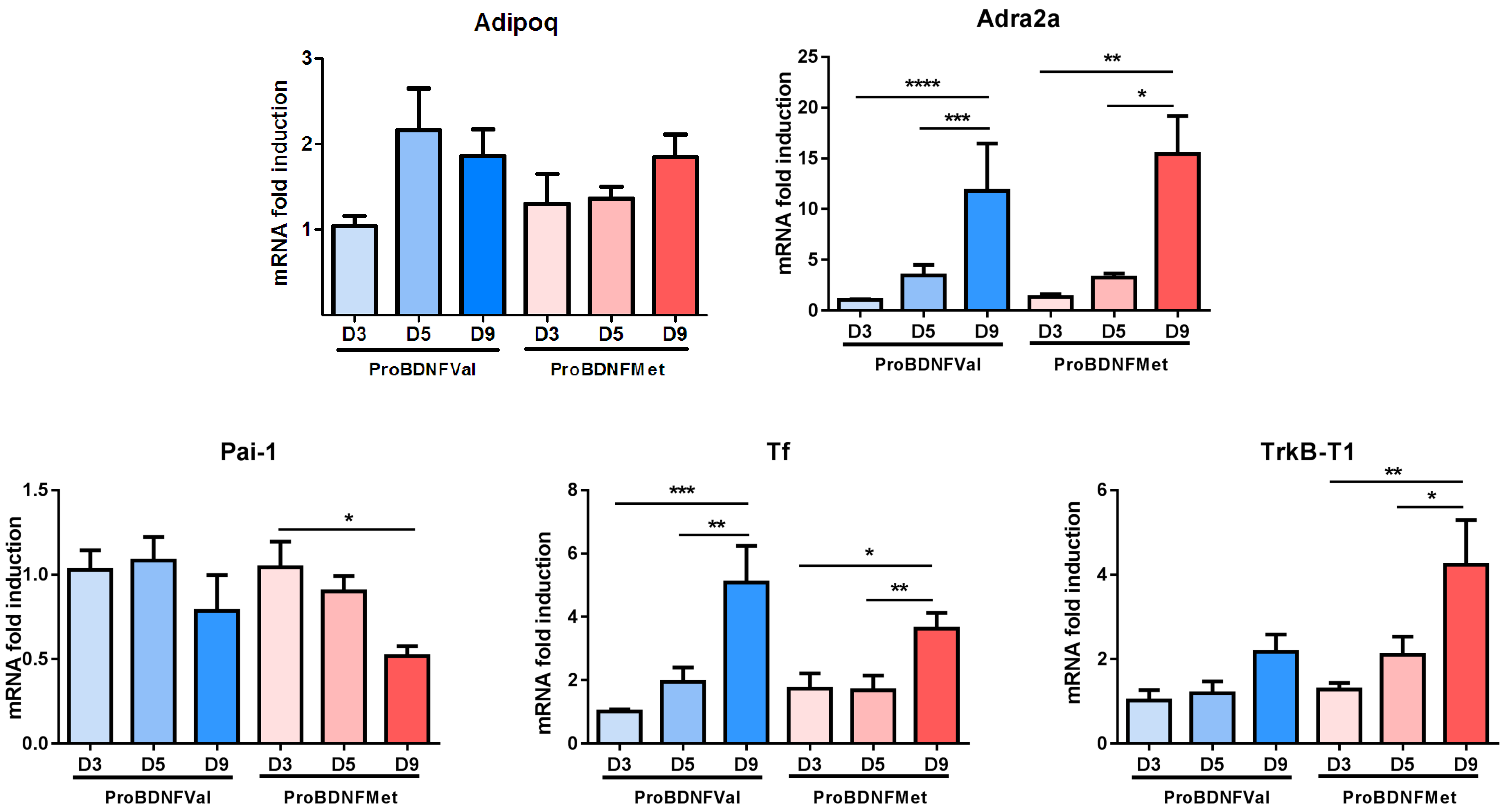

Figure S3. Evaluation of the functional relevance of BDNF Val66Met protein on C3H10T1/2 cells adipogenic differentiation.
mRNA levels of previously analyzed genes. Data are expressed as mean ± SEM. n = 5 independent experiments/group. Two-way ANOVA followed by Bonferroni post hoc analysis. * $p < 0.05$, ** $p < 0.01$, *** $p < 0.005$, **** $p < 0.001$.
