# Supplementary material for: Physical Exercise Affects Adipose Tissue Profile and Prevents Arterial Thrombosis in BDNF Val66Met Mice
Source: Cells. 2019 Aug 11;8(8):875. doi: 10.3390/cells8080875 (PMC6721716; doi:10.3390/cells8080875)
Supplement: Supplementary file 1 [file cells-08-00875-s001.zip › Supplementary/Table S1.pdf]

Table S1. Primers sequences of the analyzed genes.

**Gene**	**Sequence**	
Gapdh	fwd	CGTGCCGCCTGGAGAAACC
	rev	TGGAAGAGTGGGAGTTGCTGTTG
Pparγ	fwd	GCCCTTTGGTGACTTTATGGA
	rev	GCAGCAGGTTGTCTTGGATG
C/ebp-α	fwd	AGAGCCGAGATAAAGCCAAAC
	rev	TCATTGTCACTGGTCAACTCC
C/ebp-β	fwd	CCCCGCGTTCATGCA
	rev	CAGTCGGGCTCGTAGT
Adipoq	fwd	AGGCATCCCAGGACATC
	rev	CCTGTCATTCCAACATCTCC
Fabp4	fwd	GGCGTGGAATTCGATGAA
	rev	GCTTGTCACCATCTCGTT
Adra2a	fwd	AAAACCTCTTCCTGGTGTCC
	rev	CGCTTCAGGTTGTACTCGAT
Il-6	fwd	AAGACAAAGCCAGAGTCCTTCAGAG
	rev	GTCTTGGTCCTTAGCCACTCCTTC
Mcp-1	fwd	CTTCTGGGCCTGCTGTTCA
	rev	CCAGCCTACTCATTGGGATCA
Tnf-a	fwd	TGCCTATGTCTCAGCCTCTTC
	rev	GAGGCCATTTGGGAACTTCT
Tgf-β	fwd	TACTATGCTAAAGAGGTCACCC
	rev	CTTCCCGAATGTCTGACGTATTG
Pai-1	fwd	GTCTTTCCGACCAAGAGCAG
	rev	ATCACTTGGCCCATGAAGAG
Tf	fwd	CCCAAACCCGTCAATCAAGTC
	rev	CCAAGTACGTCTGCTTCACAT
CD163	fwd	GGGTCATTCAGAGGCACACTG
	rev	CTGGCTGTCCTGTCAAGGCT
CD80	fwd	CTGGGAAAAACCCCCAGAAG
	rev	TGACAACGATGACGACGACTG
Sorl1	fwd	GTGTGAGGACGGCGAGGCAT
	rev	GGTGGACTGCTGCCTCTGGTCA
Sirt1	fwd	AGCAGGTTGCAGGAATCCAA
	rev	CACGAACAGCTTCACAATCAACTT
Bdnf	fwd	TCGTTCCTTTCGAGTTAGCC
	rev	TTGGTAAACGGCACAAAAC
Trk Full	fwd	CTCAAGTTGGCGAGACATTCCAAG
	rev	GGGGGTTTTCAATAACAGGAATCT
TrkB-T1	fwd	TGGTGATGTTGCTCCTGCTC
	rev	CCCATCCAGTGGGATCTTATGA
