# Supplementary material for: Physical Exercise Affects Adipose Tissue Profile and Prevents Arterial Thrombosis in BDNF Val66Met Mice
Source: Cells. 2019 Aug 11;8(8):875. doi: 10.3390/cells8080875 (PMC6721716; doi:10.3390/cells8080875)
Supplement: Supplementary file 1 [file cells-08-00875-s001.zip › Supplementary/Table S2.pdf]

Table S2. F and P values referred to each graph analyzed by Two-way ANOVA or Three-way ANOVA. The heading of each table refers to the Figure number in the text.

Figure 4

% ingWAT mass/Body weight	F value	P value
Interaction	F= 0,9824	P=0,3384
Treatment	F= 0,0003243	P=0,9859
Genotype	F= 19,49	P=0,0006
% epiWAT mass/Body Weight		
Interaction	F= 0,1144	P=0,7402
Treatment	F= 0,1526	P=0,7020
Genotype	F= 267	P<0,0001

Figure 5

Adipoq	F value	P value
Interaction	F= 6,505	P=0,0201
Treatment	F= 2,867	P=0,1076
Genotype	F= 18,18	P=0,0005
Mcp-1		
Interaction	F= 10,06	P=0,0056
Treatment	F= 7,033	P=0,0168
Genotype	F= 5,112	P=0,0372
Il-6		
Interaction	F= 30,71	P<0,0001
Treatment	F= 15,47	P=0,0012
Genotype	F= 73,71	P<0,0001
Tnf-alpha		
Interaction	F= 6,05	P=0,0257
Treatment	F= 15,98	P=0,0010
Genotype	F= 127,7	P<0,0001
Tgf-beta		
Interaction	F= 12,65	P=0,0026
Treatment	F= 6,892	P=0,0184
Genotype	F= 6,411	P=0,0222
Pai-1		
Interaction	F= 12,83	P=0,0020
Treatment	F= 7,189	P=0,0148
Genotype	F= 5,342	P=0,0322
CD80		
Interaction	F= 27,77	P<0,0001
Treatment	F= 24,06	P<0,0001
Genotype	F= 66,53	P<0,0001
CD163		
Interaction	F= 15,92	P=0,0007
Treatment	F= 12,17	P=0,0023
Genotype	F= 21,14	P=0,0002

Bdnf		
Interaction	F= 14,78	P=0,0011
Treatment	F= 0,07416	P=0,7883
Genotype	F= 0,01268	P=0,9115
Sorl1		
Interaction	F = 6,777	P=0,0208
Treatment	F= 37,77	P<0,0001
Genotype	F= 13,21	P=0,0027

Figure 6

Platelets	F value	P value
Interaction	F= 0,3116	P=0,5829
Treatment	F= 18,15	P=0,0004
Genotype	F= 14,64	P=0,0011
Leukocytes		
Interaction	F= 9,286	P=0,0064
Treatment	F= 45,86	P<0,0001
Genotype	F= 36,56	P<0,0001
% platelet/leukocyte aggregates		
Interaction	F= 8,605	P=0,0082
Treatment	F= 24,01	P<0,0001
Genotype	F= 10,03	P=0,0048
Trombosi		
Time	F= 10,89	P<0,0001
Treatment	F= 690	P<0,0001
Genotype	F= 551,1	P<0,0001
Time x Treatment	F= 3,89	P<0,0001
Time x Genotype	F= 2,293	P=0,0001
Treatment x Genotype	F= 302,7	P<0,0001
Treatment x Genotype x Time	F= 1,574	P=0,0277

Figure S2

Pparg	F value	P value
Interaction	F= 0,09276	P=0,7642
Treatment	F= 1,42	P=0,2489
Genotype	F= 13,9	P=0,0015
C/ebp-alpha		
Interaction	F= 0,1999	P=0,6596
Treatment	F= 0,03827	P=0,8469
Genotype	F= 36,77	P<0,0001
C/ebp-beta		
Interaction	F= 0,0166	P=0,8988
Treatment	F= 0,4575	P=0,5065
Genotype	F= 45,32	P<0,0001
Fabp4		
Interaction	F= 0,04116	P=0,8414
Treatment	F= 0,02462	P=0,8770
Genotype	F= 0,05556	P=0,8162
Tf		
Interaction	F= 1,788	P=0,1962
Treatment	F= 1,735	P=0,2027
Genotype	F= 6,051	P=0,0231
Adra2a		
Interaction	F= 0,3135	P=0,5833
Treatment	F= 2,022	P=0,1742
Genotype	F= 5,874	P=0,0276
Sirt1		
Interaction	F= 0,006876	P=0,9347
Treatment	F= 0,2504	P=0,6223
Genotype	F= 40,36	P<0,0001
TrkB-Full		
Interaction	F= 0,223	P=0,6432
Treatment	F= 1,656	P=0,2165
Genotype	F= 0,01476	P=0,9048

TrkB-T1		
Interaction	F= 2,281	P=0,1505
Treatment	F= 0,2184	P=0,6465
Genotype	F= 0,0005985	P=0,9808
